# Age‐Related Genetic Causal Association Between Asthma and Delirium: A Bidirectional Two‐Sample Mendelian Randomization

**DOI:** 10.1002/brb3.71198

**Published:** 2026-01-28

**Authors:** Xiaopeng Wang, Guangquan Guo, Jinfeng Liu

**Affiliations:** ^1^ Department of Anesthesiology Weifang People's Hospital Weifang China

**Keywords:** age, asthma, causal analysis, delirium, Mendelian randomization, single nucleotide polymorphisms

## Abstract

**Introduction:**

Despite observational studies suggesting a link between asthma and delirium, establishing a definitive causal relationship has been challenging. This study aims to investigate the genetic causality between these conditions, with a particular focus on age‐related genetic associations using data from large‐scale genome‐wide association studies (GWASs).

**Methods:**

Bidirectional two‐sample Mendelian Randomization (MR) analyses were performed using GWAS summary statistics to evaluate the genetic association between asthma (overall, adult‐onset, childhood‐onset, and age at asthma diagnosis) and delirium. The inverse variance‐weighted (IVW) method was the primary analytic approach, supplemented by weighted median, weighted mode, and MR‐Egger methods. Multivariable Mendelian Randomization (MVMR) analyses were conducted to adjust for major confounders, including smoking, body mass index (BMI), alcohol intake, and education. Sensitivity analyses included MR‐Egger regression, MR‐PRESSO outlier tests, Cochran's Q for heterogeneity, and leave‐one‐out analyses.

**Results:**

The primary MR analyses found no evidence of a significant genetic causal relationship between asthma and delirium in either direction (asthma to delirium: IVW OR = 1.02, 95% CI: 0.79–1.32, *p* = 0.86; delirium to asthma: IVW OR = 1.05, 95% CI: 0.99–1.11, *p* = 0.13). Age‐stratified MR analyses for adult‐onset and childhood‐onset asthma, as well as age at asthma diagnosis, also showed no significant associations with delirium risk. In MVMR analyses adjusting for smoking, BMI, alcohol intake, and education, the direct effects of asthma and its subtypes on delirium, and of delirium on asthma phenotypes, remained non‐significant (all *p* > 0.05). Sensitivity analyses confirmed the robustness of these results, with no evidence of pleiotropy or heterogeneity.

**Conclusion:**

These comprehensive bidirectional MR and MVMR analyses do not support a genetic causal association between asthma and delirium, even after adjusting for key confounders and across age‐related asthma subtypes. These findings suggest that previously observed associations may be attributable to non‐genetic factors. Future studies integrating clinical and biomarker data are warranted to further explore these relationships.

## Graphical abstract

Mendelian randomization (MR) study finds no genetic evidence supporting a causal relationship between asthma and delirium across all age groups, challenging previous observational associations.

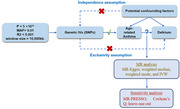

**Abbreviations**
GWASs: genome‐wide association studies
ICUs: Intensive Care Units
IVs: instrumental variables
IVW: inverse variance‐weighted
MR: Mendelian Randomization
MVMR: Multivariable Mendelian Randomization
SNPs: single nucleotide polymorphisms

## 1 Introduction

Delirium is characterized by a rapid onset of cognitive impairment, including marked disturbances in attention, consciousness, and cognition (Yang et al. 2023; Zhang et al. 2023). It manifests clinically through a range of symptoms such as perceptual distortions, cognitive disarray, delusions, hallucinations, and behavioral changes. The incidence of delirium is particularly high in older adults, with age being a major predisposing factor (Rudy and Saller 2023; Xiao et al. 2023). Age‐related changes in brain physiology, cognitive decline, and the increased prevalence of comorbidities, such as sensory deficits and frailty, heighten the risk of delirium in elderly individuals (Inouye et al. 2014; Kim et al. 2023). These vulnerabilities are further exacerbated in settings like Intensive Care Units (ICUs), where severe illnesses and environmental factors increase delirium risk (Marcantonio 2017). Additionally, chronic diseases common in aging populations, such as asthma, may indirectly elevate the likelihood of delirium through mechanisms like hypoxemia and systemic inflammation (Shin et al. 2023; Urbánek et al. 2023).

Asthma, a chronic inflammatory respiratory condition, can further complicate this scenario. Asthma not only directly affects respiratory function but also indirectly influences cerebral oxygenation and neurological function (Ntontsi et al. 2021; Miller et al. 2021). Severe asthma exacerbations can lead to hypoxemia, a critical reduction in oxygen supply to the brain, potentially triggering delirium (Mims 2015). Moreover, medications used to treat asthma, such as β2 agonists and corticosteroids, have been shown to affect the central nervous system, with prolonged or excessive use heightening the risk of delirium (Gans and Gavrilova 2020; Kwah and Peters 2019; Boulet and Boulay 2011). In elderly patients with preexisting cognitive deficits, the added stress of acute asthma attacks may provoke physiological and psychological responses that are conducive to delirium (Jones et al. 2018; Papadopoulos et al. 2022; Agache et al. 2022). Recent clinical observations suggest that asthmatic patients, particularly those of advanced age, frequently exhibit symptoms of delirium during severe attacks. Hospital data indicate that elderly asthmatic patients have a higher incidence of delirium compared to younger individuals and other patient groups (Wu et al. 2019; Ely et al. 2004). This highlights the complex interplay between age‐related vulnerabilities and the pathophysiology of both asthma and delirium, warranting a deeper investigation into their potential genetic associations.

To better understand this relationship, Mendelian Randomization (MR) offers a robust framework for investigating causal relationships while overcoming the inherent biases of observational studies (Birney 2022). MR is a genetic epidemiological method that uses genetic variants—typically single nucleotide polymorphisms (SNPs) as instrumental variables to infer causal effects between exposures and outcomes (Bowden and Holmes 2019; Sekula et al. 2016). Because genetic variants are randomly assorted at conception, MR analyses are less susceptible to confounding and reverse causation, thus providing more reliable evidence for causality. This approach has been successfully applied in various fields, such as elucidating the causal impact of obesity on osteoarthritis (Yang, 2023 #4443), investigating the role of the interleukin‐6 receptor in inflammatory bowel disease (Li et al., 2024), and clarifying potential causal relationships between gut microbiota and neuropsychiatric disorders (Li et al., 2023). Unique advantages of the MR method include its ability to mimic randomized controlled trials in the context of population genetics and its capacity to strengthen causal inference when randomized trials are impractical or unethical. These strengths make MR an increasingly valuable tool in modern epidemiological research, especially for complex diseases.

In our study, we applied a bidirectional two‐sample MR approach to explore the potential genetic causal relationship between asthma and delirium, with particular emphasis on understanding whether age modifies this association. Specifically, we aimed to elucidate whether delirium could act as a precipitant for asthma exacerbations or vice versa and whether age influences this genetic interaction. Our investigation seeks to deepen the understanding of the genetic interplay between these conditions, potentially guiding more effective clinical interventions, especially for elderly patients who are at a higher risk of both asthma and delirium.

## 2 Materials and Methods

### 2.1 Study Design Overview

**FIGURE 1:**
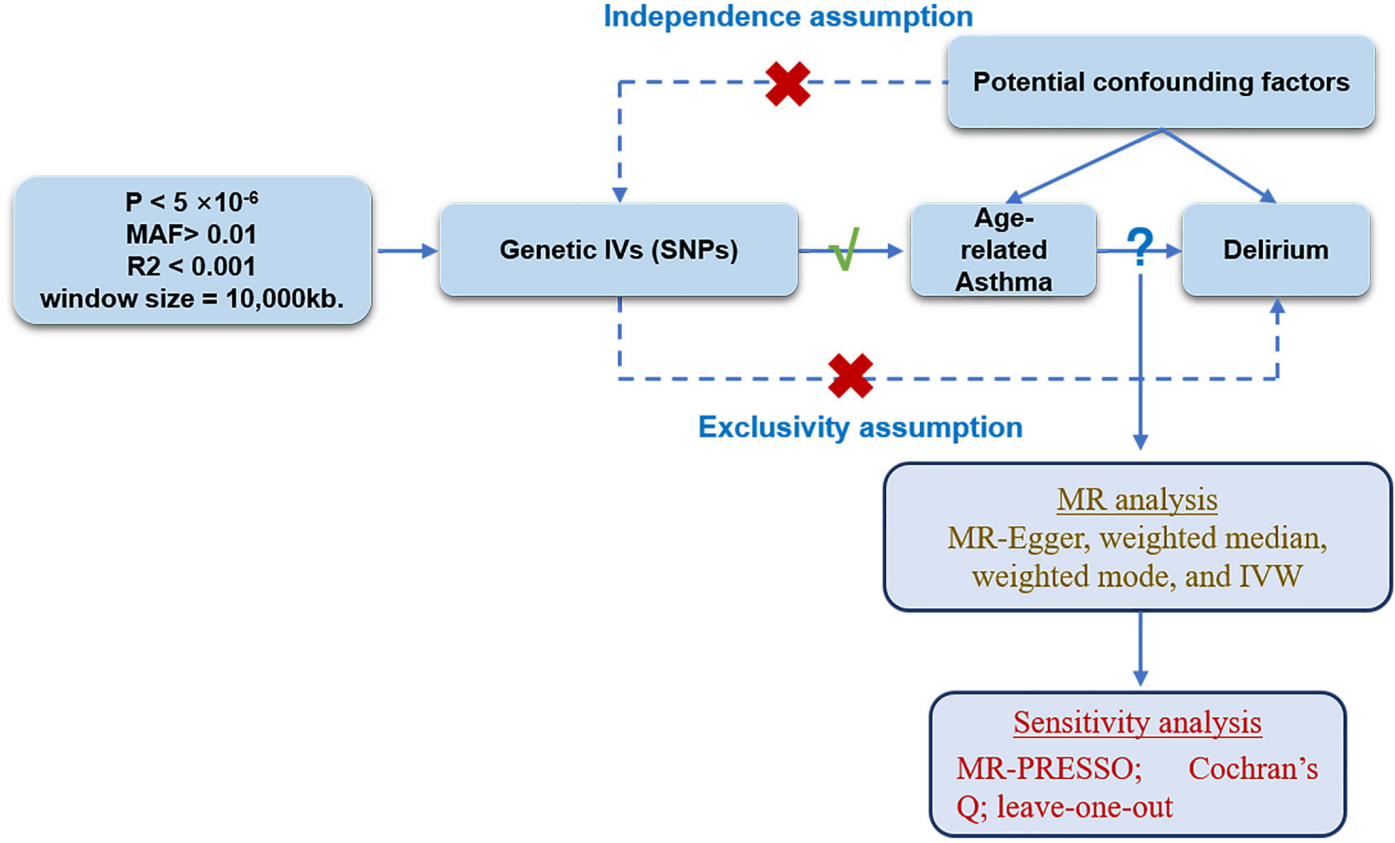
**Flowchart of the study. Abbreviations**: GWAS, genome wide association study; MR, Mendelian randomization; SNP, single nucleotide polymorphism.

In this study, we utilized a two‐sample MR approach, based on publicly available GWAS, to explore the potential causal relationship between asthma (exposure) and delirium (outcome). We employed single nucleotide polymorphisms (SNPs) associated with asthma as instrumental variables (IVs). Our MR analysis is grounded on three key assumptions, illustrated in Figure 1: (1) Relevance Assumption: The SNPs must be strongly associated with asthma, ensuring they are valid instruments for influencing delirium through asthma. (2) Independence Assumption: The SNPs should not be linked with any confounders that affect both asthma and delirium, minimizing the risk of spurious associations. (3) Exclusion Restriction Assumption: The SNPs' impact on delirium should occur only through their effect on asthma, without other pathways involved. Figure 1 presents the flowchart of the MR study framework, detailing SNP selection from GWAS data, validation against the MR assumptions, and the analysis process to assess their impact on delirium risk. This approach aims to ensure that any observed genetic associations between asthma and delirium are indicative of a causal relationship.

### 2.2 Data Sources

#### 2.2.1 Exposure Data of Asthma

The exposure data for asthma was extracted from the GWAS dataset published in Nature Communications (Guindo‐Martínez et al. 2021). This dataset included a total of 56,637 European ancestry individuals, comprising 9209 asthma cases and 47,428 controls. The GWAS summary‐level data of asthma was made available through the IEU open GWAS project. In addition to this primary asthma dataset, we also incorporated specific data for adult‐onset asthma and childhood‐onset asthma from separate GWAS studies. The dataset for adult‐onset asthma included 26,582 cases and 300,671 controls, while the dataset for childhood‐onset asthma consisted of 13,962 cases and 300,671 controls. Moreover, we used a dataset on the age of asthma diagnosis, which included 47,222 individuals. These datasets allowed us to conduct a more detailed analysis of different asthma subtypes and their potential links to delirium.

#### 2.2.2 Outcome Data of Delirium

For the outcome, we used GWAS summary statistics for delirium, specifically excluding cases induced by alcohol or other psychoactive substances (Kurki et al. 2022). The data was sourced from the FinnGen biobank and the IEU Open GWAS project. The delirium dataset included a total of 210,756 European ancestry individuals, with 1269 cases and 209,487 controls. Further details about the dataset can be accessed via the FinnGen R8 release at https://r8.risteys.finngen.fi/.

### 2.3 Data Sources for Multivariable Mendelian Randomization (MVMR) Analysis

To further address the impact of potential confounding factors, we conducted a multivariable Mendelian randomization (MVMR) analysis. The GWAS summary statistics for the following traits were incorporated as potential confounders in the MVMR framework: smoking behavior (GWAS ID: GCST90043686, *n* = 7577/105,807), body mass index (BMI, GWAS ID: GCST90025994, *n* = 457,756), alcohol intake (GWAS ID: GCST90041820, *n* = 156,663/75,922), and educational attainment (GWAS ID: GCST90011874, *n* = 510,795). The BMI dataset used in the present analysis was updated to GCST90025994 to ensure the largest available sample size and coverage.

### 2.4 Genetic Instruments Selection and Harmonization

For the selection and harmonization of IVs in this study, we established a rigorous set of criteria to ensure the robustness and validity of our MR analysis. The steps involved in selecting the appropriate IVs are as follows: (1) Initial Selection: We begin by identifying SNPs that are significantly associated with the exposure of interest (asthma or delirium, depending on the direction of the MR analysis) across the entire genome. SNPs are chosen based on a stringent *p*‐value threshold of less than 5 × 10^−6^. (2) Minor Allele Frequency (MAF) Screening: SNPs are further screened to ensure they have a minimum MAF greater than 0.01. This criterion helps ensure sufficient genetic variation and statistical power in subsequent analyses. (3) Linkage Disequilibrium (LD) Filtering: To avoid redundancy and the potential inflation of effects due to closely linked SNPs, we apply an LD threshold. SNPs are only included if they have an *R* ^2^ value of less than 0.001 within a window size of 10,000 kb (Burgess et al. 2019). (4) Substitution for Missing IVs: If an IV is not available in the summary data for the outcome, we identify alternative SNPs that are in high LD (*R* ^2^ > 0.8) with the missing IV. These substitute SNPs are used to maintain the integrity and continuity of the analysis. (5) Instrument Strength Evaluation: To determine the proportion of variability in the exposure factor that our selected instrumental variables explain, we employed the *R* ^2^ statistic. The association strength between the instrumental variables and the exposure risk was evaluated using the F‐statistic. The robustness of each SNP as an instrumental variable was determined by calculating its F‐statistic with the formula: *F* = *R* ^2^ × (N − 2) / (1 − *R* ^2^), where *R* ^2^ represents the proportion of the exposure variance explained by the SNP. An F‐statistic greater than 10 is considered robust, indicating a strong and valid instrument (Lv et al. 2021; Smith and Ebrahim 2003). These criteria are designed to minimize potential biases and maximize the causal inference strength from the genetic associations observed between asthma (or delirium, in reverse analyses) and the outcomes of interest. This approach ensures that only the most reliable genetic instruments are used in our analysis, enhancing the validity of our findings.

#### 2.4.1 Statistical Power Calculation

To evaluate whether our study had sufficient power to detect modest genetic effects, we performed post hoc statistical power calculations using established formulas for MR studies. The calculations incorporated the sample sizes of the GWAS datasets, the proportion of variance explained (*R* ^2^) by the selected instrumental variables, and the estimated effect sizes.

### 2.5 Statistical Analyses

#### 2.5.1 MR Analysis

To explore the potential causal relationships between asthma and delirium, we primarily utilized the MR approach. Our main analytical method was the random‐effects inverse‐variance weighted (IVW) method, which is particularly robust for detecting causal relationships in the absence of pleiotropy (Ference 2022). In addition to the IVW method, we employed alternative methods to ensure robustness, including the weighted median, weighted mode, and MR‐Egger methods (Figure 1).

### 2.6 Sensitivity Analysis

The potential for directional horizontal pleiotropy, which could bias our estimates, was assessed using the MR‐Egger intercept tests and MR‐PRESSO. We also assessed heterogeneity in the instrumental variable effects using Cochran's *Q* statistics and inspected funnel plots for asymmetry (Liu et al. 2019; Cao et al. 2022). To ensure the robustness of our findings, we conducted sensitivity analyses, including a leave‐one‐out approach. This method involves sequentially removing one SNP at a time from the analysis to examine if our results were disproportionately influenced by any single SNP. Additionally, to address the possibility of reverse causation—where delirium might influence the risk of asthma—we performed reverse causality analysis. This step is critical for validating the directionality and causality inferred from our MR analysis. All statistical analyses were performed using TwoSampleMR packages in R (version 4.0.5; www.r‐project.org) and Stata 16 software (Stata Corp., College Station, TX).

### 2.7 MVMR Procedure

The MVMR analysis was performed following standardized procedures to ensure the validity of the results. IVs for each exposure and confounder were initially selected using a genome‐wide significance threshold of *p* < 5 × 10^−^^8^. For the smoking phenotype (GCST90043686), due to an insufficient number of genome‐wide significant SNPs, a more relaxed threshold of *p* < 5 × 10^−^^6^ was applied. After primary SNP selection, we excluded all variants with an MAF ≤ 0.01 to maintain sufficient genetic variation and statistical power. LD pruning was then performed separately for each trait using an *R* ^2^ threshold of less than 0.001 and a window size of 10,000 kb in order to remove correlated variants and ensure the independence of genetic instruments.

After obtaining LD‐pruned SNPs for each trait, we generated the union set of all SNPs across exposures and confounders, removed duplicates, and performed an additional round of LD pruning on the combined pool to ensure stringent independence among all included instruments. Palindromic SNPs were subsequently removed for harmonization, and only non‐palindromic SNPs present in both the exposure and each confounder were retained as valid instruments for the MVMR analysis. The final set of SNPs was thus used to estimate the direct effect of asthma on delirium while simultaneously controlling for smoking, body mass index (BMI), alcohol intake, and educational attainment. All MVMR analyses were conducted using the MVMR and TwoSampleMR packages in R (version 4.0.5; www.r‐project.org).

## 3 Results

### 3.1 Bidirectional MR Analysis of Asthma and Delirium

#### 3.1.1 Asthma as Exposure

In this study, we identified a total of 19 asthma‐related IVs for the primary analysis. The mean F‐statistic for these IVs was 26.37, with a range between 19.15 and 60.80, indicating sufficient instrument strength. Three SNPs were not matched in the summary data for delirium. In the supplementary analysis, 56 IVs were identified for adult‐onset asthma, with an average F‐statistic of 66.45 (range: 30.05–312.96). Fifteen SNPs were not matched in the summary data. For childhood‐onset asthma, 105 IVs were selected, with a mean F‐statistic of 80.17 (range: 29.79–521.61), and 14 SNPs were unmatched. One palindromic or weak IV (rs11121240) was excluded. Additionally, 23 IVs related to the age of asthma diagnosis were identified, with a mean F‐statistic of 67.66 (range: 30.16–288.97).

Statistical power analyses for asthma‐related MR analyses indicated that, despite the modest effect sizes, the power to detect a causal association was generally low for individual SNPs (typically <10%), except for a subset of SNPs with larger effect sizes or higher explained variance (Supplementary Table S1). For age of asthma diagnosis, all IVs demonstrated 100% power due to the relatively high R^2^ and large sample size. For adult‐onset and childhood‐onset asthma, most IVs demonstrated low power (<10%), with only a few reaching higher levels. The limited power may partially explain the absence of significant associations in the MR analyses.

**FIGURE 2:**
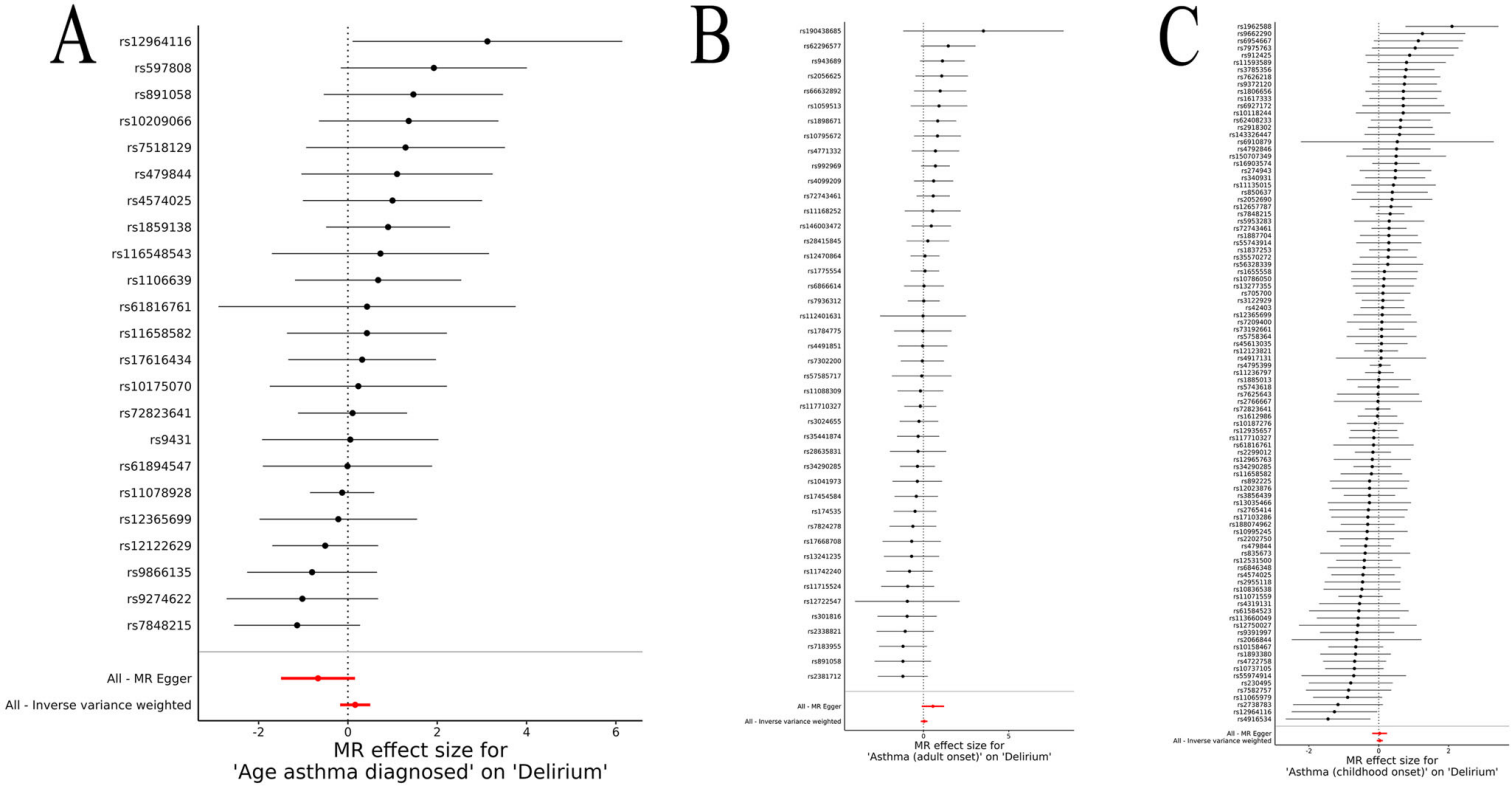
**Forest plots of single‐SNP MR for asthma on delirium. (A)** Age of asthma diagnosis to delirium; **(B)** Adult‐onset asthma to delirium; and **(C)** Childhood‐onset asthma to delirium.

The MR analysis did not reveal a statistically significant association between genetically predicted asthma, adult‐onset asthma (Figure 2B), childhood‐onset asthma (Figure 2C), or the age of asthma diagnosis (Figure 2A) and the risk of delirium. The scatter plots of SNP effects for asthma on delirium (Supplementary Figure S1) did not demonstrate a significant causal relationship. Forest plots (Figure 2) and funnel plots (Supplementary Figure S2) further confirmed these findings, showing no significant associations for any of the asthma subtypes. Additionally, leave‐one‐out sensitivity analysis (Supplementary Figure S3) identified one significant outlier (rs11078928). After removing this outlier, the MR analysis did not reveal a statistically significant association between genetically predicted asthma, adult‐onset asthma, childhood‐onset asthma, or the age of asthma diagnosis and the risk of delirium. For overall asthma, the odds ratio (OR) was 1.02 (95% CI: 0.79–1.32, *p* = 0.86) using the IVW method. Similar results were obtained using the MR‐Egger, weighted median, and weighted mode methods. Both adult‐onset and childhood‐onset asthma showed no significant association with delirium risk (adult‐onset: OR = 1.04, 95% CI: 0.85–1.26, *p* = 0.707; childhood‐onset: OR = 1.03, 95% CI: 0.95–1.12, *p* = 0.508). Additionally, the age of asthma diagnosis was not significantly associated with delirium (OR = 1.18, 95% CI: 0.84–1.65, *p* = 0.344) (Table 1). No significant pleiotropy was observed in the MR‐Egger intercepts (Supplementary Table S2). The MR‐PRESSO global test indicated no additional outliers, confirming the robustness of the results (Supplementary Table S3).

**TABLE 1:** Association between genetically predicted asthma, adult‐onset asthma, childhood‐onset asthma, and the age of asthma diagnosis with the risk of delirium, before and after outlier removal.

Exposure	Outcome	N.SNPs	Methods	OR (95% CI)	P
Before outliers removal:					
Asthma	Delirium	15	IVW	1.02 (0.79–1.32)	0.86
			MR‐Egger	0.68 (0.38–1.24)	0.23
			Weighted median	1.1 (0.77–1.59)	0.59
			Weighted mode	1.33 (0.72–2.45)	0.38
Asthma (adult onset)		44	IVW	1.04 (0.85–1.26)	0.707
			MR‐Egger	1.74 (0.91–3.34)	0.103
			Weighted median	1.02 (0.77–1.35)	0.903
			Weighted mode	0.94 (0.57–1.53)	0.792
Asthma (childhood onset)		97	IVW	1.03 (0.95–1.12)	0.508
			MR‐Egger	1.02 (0.83–1.27)	0.819
			Weighted median	1.04 (0.91–1.18)	0.584
			Weighted mode	1.04 (0.87–1.24)	0.656
Age asthma diagnosed		23	IVW	1.18 (0.84–1.65)	0.344
			MR‐Egger	0.51 (0.22–1.17)	0.128
			Weighted median	1.00 (0.61–1.63)	0.996
			Weighted mode	0.95 (0.52–1.73)	0.869
After outliers removal:					
Asthma	Delirium	15	IVW	1.02 (0.79–1.32)	0.86
			MR‐Egger	0.68 (0.38–1.24)	0.23
			Weighted median	1.1 (0.77–1.59)	0.59
			Weighted mode	1.33 (0.72–2.45)	0.38
Asthma (adult onset)		44	IVW	1.04 (0.85–1.26)	0.707
			MR‐Egger	1.74 (0.91–3.34)	0.103
			Weighted median	1.02 (0.76–1.35)	0.903
			Weighted mode	0.94 (0.55–1.58)	0.805
Asthma (childhood onset)		97	IVW	1.03 (0.95–1.12)	0.508
			MR‐Egger	1.02 (0.83–1.27)	0.819
			Weighted median	1.04 (0.92–1.17)	0.554
			Weighted mode	1.04 (0.88–1.23)	0.647
Age asthma diagnosed		22	IVW	1.27 (0.87–1.87)	0.214
			MR‐Egger	0.49 (0.18–1.37)	0.188
			Weighted median	1.20 (0.69–2.07)	0.516
			Weighted mode	1.23 (0.50–3.05)	0.662

Sensitivity analysis.

### 3.2 MVMR Analysis

**FIGURE 3:**
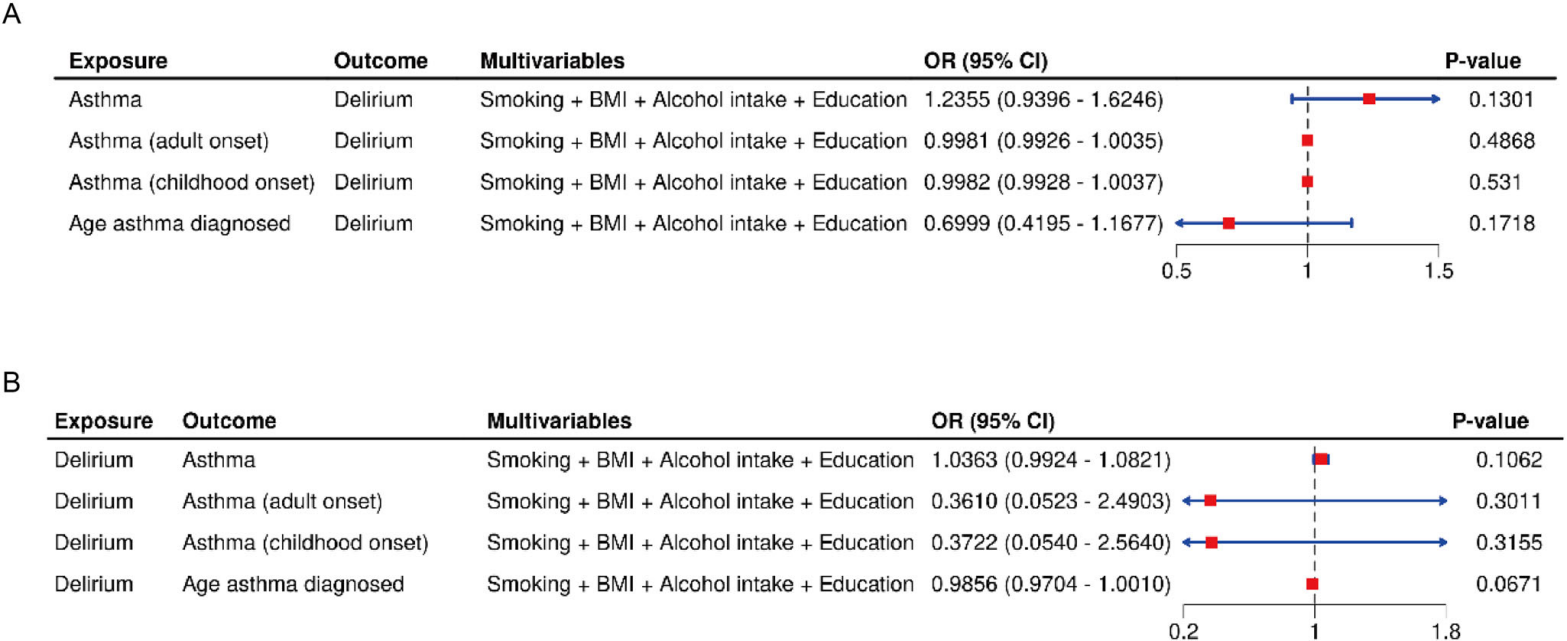
**Multivariable Mendelian Randomization (MVMR) Analyses of Asthma and Delirium**. (A) Forest plot of the direct effects of asthma and its subtypes on delirium after adjustment for smoking, body mass index (BMI), alcohol intake, and education; and **(B)** Forest plot of the direct effects of delirium on asthma and its subtypes after adjustment for smoking, BMI, alcohol intake, and education.

To further account for potential confounding from lifestyle and socioeconomic factors, we conducted MVMR analyses adjusting for smoking, BMI, alcohol intake, and education. After adjusting for these confounders, none of the asthma phenotypes demonstrated a significant direct effect on delirium risk (all *p* > 0.05). Specifically, the direct effect ORs (95% CI, *p*‐value) were as follows: asthma—1.24 (0.94–1.62, *p* = 0.1301); adult‐onset asthma—1.00 (0.99–1.00, *p* = 0.4868); childhood‐onset asthma—1.00 (0.99–1.00, *p* = 0.531); age at asthma diagnosis—0.70 (0.42–1.17, *p* = 0.1718) (Figure 3A). These findings are consistent with the results from the univariable MR analysis, further supporting the absence of a direct genetic causal relationship between asthma and delirium, even after controlling for major confounders.

#### 3.2.1 Delirium as Exposure

In the reverse MR analysis, we identified six IVs associated with delirium, with a mean F‐statistic of 27.37 (range: 21.64–43.43). All SNPs were available in the summary data for asthma, adult‐onset asthma, and childhood‐onset asthma outcomes. However, for the age of asthma diagnosis outcome, one SNP (rs7571327) was not matched in the summary data.

As shown in Supplementary Table S1, the power of the MR analyses ranged from 5% to 41%. The analysis of age at asthma diagnosis on delirium had the highest power (41%), while other asthma‐related exposures showed low power (5%). In the reverse analyses (delirium as exposure), all *r* ^2^ values were 0.000779 with similarly low power (5%). The power for delirium and age at asthma diagnosis was NA, as the outcome was a continuous variable. Overall, the results indicate limited power, especially for the reverse MR analyses. In contrast to the asthma‐to‐delirium direction, the statistical power for the reverse direction (delirium as exposure) was limited, mainly due to the small number of delirium cases (*n* = 1269) and the modest proportion of variance explained by the available IVs. As shown in Supplementary Table S1, most individual IVs had low statistical power (<10%) to detect modest genetic effects in the reverse MR analysis. This limitation should be considered when interpreting the null findings for this direction.

**FIGURE 4:**
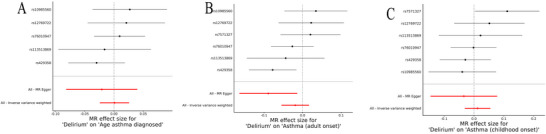
**Forest plots of single‐SNP MR for delirium on asthma. (A)** Delirium to age of asthma diagnosis; **(B)** Delirium to adult‐onset asthma; and **(C)** Delirium to childhood‐onset asthma.

The scatter plots of SNP effects for delirium on asthma (Supplementary Figure S4) showed no evidence of a genetic association with asthma risk. Forest plots (Figure 4) and funnel plots (Figure S5) confirmed the lack of significant associations for any of the asthma subtypes. The IVW method produced an OR of 1.05 (95% CI: 0.99–1.11, *p* = 0.13) for asthma, with no significant associations for adult‐onset asthma (OR = 0.98, 95% CI: 0.95–1.02, *p* = 0.296, Figure 4B) or childhood‐onset asthma (OR = 1.01, 95% CI: 0.97–1.06, *p* = 0.589, Figure 4C). The age of asthma diagnosis also showed no significant relationship with delirium (OR = 1.00, 95% CI: 0.98–1.03, *p* = 0.946, Figure 4A) (Table 2). The leave‐one‐out sensitivity analysis (Supplementary Figure S6) similarly revealed no significant associations for delirium on asthma risk. No significant pleiotropy was detected in the MR‐Egger intercept test, and heterogeneity was not observed in the IVW Q‐statistics (Supplementary Table S4). Similarly, the MR‐PRESSO analysis did not identify any outliers, confirming the robustness of these findings (Supplementary Table S5).

**TABLE 2:** Association between genetically predicted delirium and the risk of asthma, adult‐onset asthma, childhood‐onset asthma, and the age of asthma diagnosis.

Exposure	Outcome	N.SNPs	Methods	OR (95% CI)	*p*
Delirium	Asthma	6	IVW	1.05 (0.99–1.11)	0.13
			MR‐Egger	1.16 (1–1.35)	0.12
			Weighted median	1.02 (0.94–1.11)	0.58
			Weighted mode	1.02 (0.89–1.16)	0.79
	Asthma (adult onset)	6	IVW	0.98 (0.95–1.02)	0.296
			MR‐Egger	0.91 (0.85–0.99)	0.081
			Weighted median	0.99 (0.94–1.03)	0.493
			Weighted mode	1.01 (0.95–1.07)	0.867
	Asthma (childhood onset)	6	IVW	1.01 (0.97–1.06)	0.589
			MR‐Egger	0.97 (0.86–1.08)	0.582
			Weighted median	1.00 (0.95–1.05)	0.908
			Weighted mode	0.99 (0.93–1.05)	0.735
	Age asthma diagnosed	5	IVW	1.00 (0.98–1.03)	0.946
			MR‐Egger	0.98 (0.92–1.04)	0.549
			Weighted median	1.01 (0.97–1.04)	0.74
			Weighted mode	1.01 (0.97–1.06)	0.535

In the reverse MVMR analysis, after adjusting for smoking, BMI, alcohol intake, and education, the direct effects of delirium on all four asthma‐related phenotypes were also not statistically significant (all *p* > 0.05, Figure 3B). Specifically, the ORs (95% CI, p value) were for asthma—1.04 (0.99–1.08, *p* = 0.1062); adult‐onset asthma—0.36 (0.05–2.49, *p* = 0.3011); childhood‐onset asthma—0.37 (0.05–2.56, *p* = 0.3155); and age at asthma diagnosis—0.99 (0.97–1.00, *p* = 0.0671). These results further corroborate the lack of a direct genetic association between delirium and asthma when major confounders are taken into account.

### 3.3 Sensitivity Analysis

Several sensitivity analyses were conducted to evaluate the robustness of the MR findings. The leave‐one‐out analysis (Supplementary Figure S3 for asthma exposure, Supplementary Figure S6 for delirium exposure) indicated that no individual SNP had a significant influence on the overall estimate, except for the outlier rs11078928, which was removed in the final analysis. The MR‐Egger regression showed no significant evidence of horizontal pleiotropy across most analyses, with the exception of the initial analysis between age of asthma diagnosis and delirium, which was corrected after removing the outlier. The MR‐PRESSO global test detected no outliers, confirming the robustness of the results (Supplementary Table S3 for asthma exposure and Supplementary Table S5 for delirium exposure).

Overall, both univariable and multivariable MR analyses consistently indicated no significant direct genetic causal association between asthma and delirium, nor in the reverse direction, even after comprehensive adjustment for smoking, BMI, alcohol intake, and education.

## 4 Discussion

Our comprehensive bidirectional and multivariable MR analyses did not reveal a significant genetic causal relationship between asthma—including adult‐onset asthma, childhood‐onset asthma, and age at asthma diagnosis—and delirium. This absence of association persisted even after adjusting for key confounders such as smoking, body mass index, alcohol intake, and education. These findings indicate that asthma, regardless of age of onset or subtype, is not a genetically independent risk factor for delirium. This contrasts with some earlier clinical observations that reported a higher incidence of delirium among patients with asthma, especially during acute exacerbations or in older adults (Ely et al. 2004; Rhyou and Nam 2021). The lack of a genetic causal link in our study suggests that previously observed associations may be driven by non‐genetic or environmental factors, warranting further investigation into alternative mechanisms.

While asthma is a chronic inflammatory disease primarily driven by immune‐related genes and environmental exposures like allergens (Ntontsi et al. 2021; Mims 2015; Khurana and Jarjour 2019), delirium's pathophysiology is more complex. Delirium involves neuroinflammation, neurotransmitter imbalances, and acute disruptions in brain function (Gandler et al. 2023; Brummel et al. 2024). The genetic pathways underlying these conditions are distinct, which may explain why our MR analysis, based on genetic instruments, did not identify a causal relationship between asthma and delirium. The non‐overlapping genetic basis of these diseases suggests that previous associations found in observational studies may have been influenced by non‐genetic factors.

Importantly, our study specifically evaluated genetic causality between asthma and delirium, but non‐genetic factors—such as medication use, comorbidities, and environmental exposures—may play a substantial role in delirium risk. Asthma medications, especially corticosteroids, are well known to have neuropsychiatric side effects, including delirium, particularly in elderly patients (Beale et al. 1951). The mechanisms may involve corticosteroid‐induced alterations in neurotransmitter function, sleep disruption, and increased susceptibility of the aging brain to medication‐induced neurotoxicity. Other asthma medications, such as β2 agonists, may also contribute to CNS side effects. Furthermore, elderly patients with asthma often have multiple comorbidities (e.g., cardiovascular disease, diabetes, or chronic kidney disease) and polypharmacy, both of which are established risk factors for delirium (Rudy and Saller 2023; Softy et al. 2023). In clinical settings, acute exacerbations of asthma may trigger hypoxemia and systemic inflammation, further increasing delirium risk. However, these factors could not be captured in our genetic analysis, highlighting a limitation of MR methodologies in assessing multifactorial clinical syndromes like delirium.

The clinical value of our study lies in correcting potential misconceptions that have arisen from earlier observational research. Although previous studies reported an association between asthma and delirium, our findings indicate that this relationship is not due to a direct genetic or biological link. Instead, the high correlation observed in clinical practice is most likely attributable to confounders, including corticosteroid use, hypoxia, ICU environment, and polypharmacy, rather than asthma itself. Therefore, when treating asthma patients—especially elderly individuals—clinicians should not focus solely on asthma as an intrinsic cause of delirium. Greater attention should be given to iatrogenic and environmental risk factors, such as optimizing medication regimens, minimizing corticosteroid side effects, improving oxygenation, and reducing unnecessary polypharmacy. This provides concrete clinical guidance, suggesting that the prevention and management of delirium in patients with asthma should prioritize modifiable risk factors rather than attributing risk solely to asthma as an underlying disease.

Despite the lack of evidence for a direct genetic link, it is important to consider shared inflammatory mechanisms. Delirium has been associated with elevated levels of pro‐inflammatory cytokines, such as interleukin‐6 (IL‐6) (Beale et al. 1951; Li et al. 2024). Similarly, asthma is characterized by chronic inflammation involving multiple mediators and cytokines, including IL‐4 and IL‐13. These shared inflammatory pathways could theoretically link asthma with an increased risk of delirium, particularly during asthma exacerbations. It is possible that systemic inflammation, acute infections, or medication effects may act as mediators between asthma and delirium. However, our MR design did not include mediation analyses or multi‐omics data, such as inflammatory biomarkers, to clarify these mechanistic links. Future MR studies incorporating inflammation‐related biomarkers as potential mediators may provide valuable mechanistic insights. In our study, stronger risk factors for delirium—such as acute systemic illnesses, infections, and medications—may have overshadowed any potential influence of chronic inflammation from asthma (Sassano‐Higgins et al. 2016; Pasrija et al. 2021).

Given that age is a well‐established risk factor for delirium, we also explored whether age‐related asthma subtypes (adult‐onset asthma, childhood‐onset asthma, and the age of asthma diagnosis) were associated with delirium risk. However, no significant associations were found in any of these subgroups. This result may be partly due to the heterogeneity of asthma across age groups. Childhood‐onset asthma is often linked to allergic mechanisms, whereas adult‐onset asthma may stem from environmental exposures, such as smoking or occupational irritants. This heterogeneity, combined with the fact that older adults often have multiple comorbidities and are more susceptible to neurodegenerative processes, may have diluted any potential association between asthma and delirium (Kim et al. 2023). Moreover, the role of age in delirium risk is complex. In older adults, delirium is more strongly associated with neurodegenerative changes, vascular pathology, and acute systemic illnesses than with chronic inflammatory conditions like asthma (Rudy and Saller 2023; Softy et al. 2023). It is possible that asthma's potential impact on delirium risk is outweighed by these other factors, especially in elderly populations, where the cumulative burden of comorbidities plays a critical role in delirium development.

Additionally, asthma manifests heterogeneously across different age groups. Childhood‐onset asthma is often closely related to allergic responses, whereas adult‐onset asthma may be associated with factors such as occupational exposures and smoking (Papadopoulos et al. 2022). This heterogeneity may dilute any potential associations between asthma and delirium in different age categories, as the underlying mechanisms of asthma in these subgroups differ significantly. Another contributing factor could be the limited statistical power in our age‐specific subgroup analyses. The reduction in sample size when stratifying by age may have increased the risk of type II errors, making it difficult to detect subtle associations. Furthermore, potential biases in the age of asthma diagnosis data, such as younger patients receiving more frequent medical interventions, could have confounded the results. It is possible that these interventions themselves may reduce the risk of delirium, further complicating the analysis.

The influence of asthma medications (such as corticosteroids) on delirium risk was not genetically proxied in our MR design. We considered the possibility of performing MVMR adjusting for medication use; however, our bidirectional MR analyses did not reveal significant direct effects, and currently available genetic proxies for medication exposures are limited. Most existing genetic instruments for drug exposure reflect prescription behavior or indication rather than direct pharmacological effects, and their instrument strength and consistency are insufficient, which would weaken the validity and statistical power of MVMR—particularly in reverse analyses with only six IVs. Moreover, when medication use is a mediator rather than a confounder, conditioning on it in MVMR may block true causal pathways, complicating the interpretation of results. Therefore, we did not pursue MVMR in this study. We acknowledge this as a limitation and emphasize the need for future MR studies with stronger genetic instruments for medication exposure and mediation analysis.

It is also important to note that the statistical power for detecting causal effects in the reverse MR analysis was limited. This was primarily due to the relatively small number of delirium cases in the GWAS dataset (*n* = 1269), as well as the modest variance explained by the available instrumental variables. As shown in our supplementary power analysis, most IVs in the reverse direction had low power (<10%) to detect modest genetic effects. Therefore, the absence of significant associations in the reverse MR analysis should be interpreted with caution, as false‐negative results due to limited power cannot be ruled out.

### 4.1 Strengths and Limitations

One of the strengths of this study is the use of MR analysis, which helps mitigate biases such as reverse causation and residual confounding that are common in observational studies. Additionally, the use of large‐scale GWAS data enhances the statistical power of the analysis. However, our study also has several limitations. Most notably, our analysis was limited to individuals of European ancestry, potentially restricting the generalizability of our findings to other populations. Future replication studies in multi‐ancestry cohorts are needed to confirm the robustness and external validity of our results. In addition, the lack of detailed clinical information—such as asthma severity, medication history, and comorbidities—may have influenced the results, and the MR method itself cannot account for the full complexity of clinical exposures and environmental factors. The reduced sample sizes in our age‐stratified analyses may have limited the power to detect subtle associations, increasing the risk of type II errors. In particular, the limited number of delirium cases in the GWAS led to reduced statistical power in the reverse MR analysis, as discussed above. Finally, the absence of mediation analyses incorporating inflammatory biomarkers or medication use represents a further limitation and an important direction for future research.

### 4.2 Future Directions

Although our study did not identify a genetic causal link between asthma and delirium, future research should explore other potential mechanisms, including gene‐environment interactions and the influence of non‐genetic factors such as medication use, systemic inflammation, and comorbidities. Larger and more diverse cohorts, with detailed clinical data—such as asthma severity, exacerbation frequency, medication use, and comorbidity profiles—are needed to clarify these complex relationships. Multi‐omics approaches, including proteomics, metabolomics, and integration of inflammatory biomarkers, could provide a more comprehensive understanding of the biological pathways connecting asthma and delirium. Mediation analyses within the MR framework, especially those incorporating biomarkers of inflammation or drug exposure, may yield valuable mechanistic insights.

Additionally, neuroimaging and biomarker studies could shed light on potential shared pathways. For example, inflammatory biomarkers such as IL‐6 and TNF‐α have been implicated in both asthma and delirium, and investigating these shared pathways could offer new insights into the relationship between these conditions (Miller et al. 2021; Birney 2022). Even in the absence of a direct genetic causal relationship, understanding these biological mechanisms could help in identifying patients at higher risk for delirium and developing more targeted prevention and intervention strategies, particularly among elderly asthma patients.

## 5 Conclusion

Our comprehensive bidirectional and multivariable MR analyses provide genetic evidence that does not support a causal relationship between asthma and delirium, including across different age‐related asthma subtypes. This lack of association persisted even after rigorous adjustment for major confounders such as smoking, body mass index, alcohol intake, and education. These findings suggest that previously observed associations in observational studies may be attributable to non‐genetic or environmental factors. Future research should focus on larger and more diverse populations and incorporate detailed clinical data, medication use, and biomarker analyses to further elucidate the complex interplay between asthma and delirium.

## Author Contributions

X. P. W. and J. F. L. carried out the studies, participated in collecting data, and drafted the manuscript. X. P. W. and J. F. L. performed the statistical analysis and participated in its design. X. P. W., J. F. L. and G. Q. G. participated in acquisition, analysis, or interpretation of data and draft the manuscript. All authors read and approved the final manuscript.

## Funding

The authors have nothing to report.

## Conflicts of Interest

The authors declare no conflicts of interest.

## Supporting information




**Supplementary Tables**: brb371198‐sup‐001‐Tables.docx.

Figure S1 **Scatter plots of SNP effects for asthma on delirium. (A)** Age of asthma diagnosis to delirium; **(B)** Adult‐onset asthma to delirium; and **(C)** Childhood‐onset asthma to delirium.
Figure S2 **Funnel plots of single‐SNP MR for asthma on delirium. (A)** Age of asthma diagnosis to delirium; **(B)** Adult‐onset asthma to delirium; and **(C)** Childhood‐onset asthma to delirium.
Figure S3 **Leave‐one‐out sensitivity analysis for asthma on delirium. (A)** Age of asthma diagnosis to delirium; **(B)** Adult‐onset asthma to delirium; and **(C)** Childhood‐onset asthma to delirium.
Figure S4 **Scatter plots of SNP effects for delirium on asthma. (A)** Delirium to age of asthma diagnosis; **(B)** Delirium to adult‐onset asthma; and **(C)** Delirium to childhood‐onset asthma.
Figure S5 **Funnel plots of single‐SNP MR for delirium on asthma. (A)** Delirium to age of asthma diagnosis; **(B)** Delirium to adult‐onset asthma; and **(C)** Delirium to childhood‐onset asthma.
Figure S6 **Leave‐one‐out sensitivity analysis for delirium on asthma. (A)** Delirium to age of asthma diagnosis; **(B)** Delirium to adult‐onset asthma; and **(C)** Delirium to childhood‐onset asthma.

## Data Availability

All data generated or analyzed during this study are included in this published article and its supplementary information files.
