# Supplementary material for: Age‐Related Genetic Causal Association Between Asthma and Delirium: A Bidirectional Two‐Sample Mendelian Randomization
Source: Brain Behav. 2026 Jan 28;16(2):e71198. doi: 10.1002/brb3.71198 (PMC12848525; doi:10.1002/brb3.71198)
Supplement: Supplementary file 1 — Supplementary Tables: brb371198‐sup‐001‐Tables.docx. [file BRB3-16-e71198-s002.docx]

**Table S1.** Statistical Power Calculations and Instrument Strength for Each Instrumental Variable Used in Mendelian Randomization Analyses of Asthma and Delirium

| Exposure | Outcone | Case | Control | Ratio | Samplesize | r2 | OR | Power |
| --- | --- | --- | --- | --- | --- | --- | --- | --- |
| Delirium | Age asthma diagnosed | 47222 |  |  | 47222 | 0.000779 | 1 | NA |
| Delirium | Asthma | 9209 | 47428 | 0.194168002 | 56637 | 0.000779 | 1.05 | 5% |
| Delirium | Asthma (adult onset) | 26582 | 300671 | 0.088408925 | 327253 | 0.000779 | 0.98 | 5% |
| Delirium | Asthma (childhood onset) | 13962 | 300671 | 0.046436138 | 314633 | 0.000779 | 1.01 | 5% |
| Age asthma diagnosed | Delirium | 1269 | 209487 | 0.006057655 | 210756 | 0.032877067 | 1.27 | "41 |
| Asthma | Delirium | 1269 | 209487 | 0.006057655 | 210756 | 0.008842221 | 1.02 | 5% |
| Asthma (adult onset) | Delirium | 1269 | 209487 | 0.006057655 | 210756 | 7.61E-03 | 1.04 | 5% |
| Asthma (childhood onset) | Delirium | 1269 | 209487 | 0.006057655 | 210756 | 0.023589924 | 1.03 | 5% |
| SNP | **exposure** | **Case** | **Control** | **Ratio** | **Samplesize** | **r2** | **OR** | **Power** |
| rs12122629 | Age asthma diagnosed | 47222 |  |  | 47222 | 0.00262 | 0.843436 | 100% |
| rs61816761 | Age asthma diagnosed | 47222 |  |  | 47222 | 0.003122 | 0.775994 | 100% |
| rs7518129 | Age asthma diagnosed | 47222 |  |  | 47222 | 0.000638 | 0.962662 | 100% |
| rs479844 | Age asthma diagnosed | 47222 |  |  | 47222 | 0.000705 | 0.963259 | 100% |
| rs61894547 | Age asthma diagnosed | 47222 |  |  | 47222 | 0.001329 | 0.896765 | 100% |
| rs12365699 | Age asthma diagnosed | 47222 |  |  | 47222 | 0.001039 | 1.063854 | 100% |
| rs597808 | Age asthma diagnosed | 47222 |  |  | 47222 | 0.000733 | 0.962424 | 100% |
| rs9431 | Age asthma diagnosed | 47222 |  |  | 47222 | 0.000812 | 1.04083 | 100% |
| rs11078928 | Age asthma diagnosed | 47222 |  |  | 47222 | 0.006082 | 1.11589 | 100% |
| rs11658582 | Age asthma diagnosed | 47222 |  |  | 47222 | 0.000942 | 0.956101 | 100% |
| rs4574025 | Age asthma diagnosed | 47222 |  |  | 47222 | 0.000784 | 0.961015 | 100% |
| rs12964116 | Age asthma diagnosed | 47222 |  |  | 47222 | 0.000836 | 0.901063 | 100% |
| rs891058 | Age asthma diagnosed | 47222 |  |  | 47222 | 0.000692 | 1.041992 | 100% |
| rs72823641 | Age asthma diagnosed | 47222 |  |  | 47222 | 0.001655 | 1.091622 | 100% |
| rs10209066 | Age asthma diagnosed | 47222 |  |  | 47222 | 0.000792 | 0.96125 | 100% |
| rs10175070 | Age asthma diagnosed | 47222 |  |  | 47222 | 0.000792 | 1.046009 | 100% |
| rs1106639 | Age asthma diagnosed | 47222 |  |  | 47222 | 0.000897 | 1.05073 | 100% |
| rs9866135 | Age asthma diagnosed | 47222 |  |  | 47222 | 0.001477 | 1.055975 | 100% |
| rs17616434 | Age asthma diagnosed | 47222 |  |  | 47222 | 0.001625 | 1.072834 | 100% |
| rs1859138 | Age asthma diagnosed | 47222 |  |  | 47222 | 0.001231 | 1.059037 | 100% |
| rs116548543 | Age asthma diagnosed | 47222 |  |  | 47222 | 0.000727 | 1.175475 | 100% |
| rs9274622 | Age asthma diagnosed | 47222 |  |  | 47222 | 0.001613 | 0.944731 | 100% |
| rs7848215 | Age asthma diagnosed | 47222 |  |  | 47222 | 0.001731 | 0.936752 | 100% |
| rs10123808 | Asthma | 9209 | 47428 | 0.1942 | 56637 | 0.000371 | 1.079541 | 3.40% |
| rs116356771 | Asthma | 9209 | 47428 | 0.1942 | 56637 | 0.000338 | 0.725372 | 3.40% |
| rs11954099 | Asthma | 9209 | 47428 | 0.1942 | 56637 | 0.000367 | 0.914649 | 3.50% |
| rs12922107 | Asthma | 9209 | 47428 | 0.1942 | 56637 | 0.000378 | 1.140463 | 3.50% |
| rs13019081 | Asthma | 9209 | 47428 | 0.1942 | 56637 | 0.000443 | 1.086811 | 3.50% |
| rs142807069 | Asthma | 9209 | 47428 | 0.1942 | 56637 | 0.000581 | 1.126174 | 4.40% |
| rs145349797 | Asthma | 9209 | 47428 | 0.1942 | 56637 | 0.000425 | 1.384604 | 8.50% |
| rs154073 | Asthma | 9209 | 47428 | 0.1942 | 56637 | 0.000519 | 1.092314 | 3.70% |
| rs17293632 | Asthma | 9209 | 47428 | 0.1942 | 56637 | 0.000424 | 1.096253 | 3.60% |
| rs2518214 | Asthma | 9209 | 47428 | 0.1942 | 56637 | 0.000373 | 0.909473 | 3.60% |
| rs252716 | Asthma | 9209 | 47428 | 0.1942 | 56637 | 0.000616 | 1.10135 | 4% |
| rs28402931 | Asthma | 9209 | 47428 | 0.1942 | 56637 | 0.000453 | 1.478225 | 10.90% |
| rs2959628 | Asthma | 9209 | 47428 | 0.1942 | 56637 | 0.000378 | 1.079325 | 3.40% |
| rs34922454 | Asthma | 9209 | 47428 | 0.1942 | 56637 | 0.000377 | 0.914852 | 3.50% |
| rs67053006 | Asthma | 9209 | 47428 | 0.1942 | 56637 | 0.000515 | 0.86965 | 4.60% |
| rs72706043 | Asthma | 9209 | 47428 | 0.1942 | 56637 | 0.000449 | 1.336536 | 7.80% |
| rs7618501 | Asthma | 9209 | 47428 | 0.1942 | 56637 | 0.00037 | 0.928439 | 3.30% |
| rs823544 | Asthma | 9209 | 47428 | 0.1942 | 56637 | 0.000393 | 1.083765 | 3.40% |
| rs9272521 | Asthma | 9209 | 47428 | 0.1942 | 56637 | 0.001072 | 1.151557 | 6% |
| rs301816 | Asthma (adult onset) | 26582 | 300671 | 0.0884 | 327253 | 9.78E-05 | 0.953193 | 3.00% |
| rs2056625 | Asthma (adult onset) | 26582 | 300671 | 0.0884 | 327253 | 0.000148 | 1.06049 | 3.20% |
| rs17668708 | Asthma (adult onset) | 26582 | 300671 | 0.0884 | 327253 | 0.000139 | 1.09563 | 3.70% |
| rs12722547 | Asthma (adult onset) | 26582 | 300671 | 0.0884 | 327253 | 0.000138 | 1.2369 | 5.80% |
| rs10795672 | Asthma (adult onset) | 26582 | 300671 | 0.0884 | 327253 | 0.000148 | 0.942709 | 3.20% |
| rs1775554 | Asthma (adult onset) | 26582 | 300671 | 0.0884 | 327253 | 0.000463 | 1.10914 | 5.40% |
| rs28415845 | Asthma (adult onset) | 26582 | 300671 | 0.0884 | 327253 | 0.000166 | 1.06962 | 3.40% |
| rs174535 | Asthma (adult onset) | 26582 | 300671 | 0.0884 | 327253 | 0.000162 | 1.06561 | 3.30% |
| rs7936312 | Asthma (adult onset) | 26582 | 300671 | 0.0884 | 327253 | 0.000317 | 0.918629 | 4.20% |
| rs1784775 | Asthma (adult onset) | 26582 | 300671 | 0.0884 | 327253 | 0.000109 | 0.946886 | 3.10% |
| rs11168252 | Asthma (adult onset) | 26582 | 300671 | 0.0884 | 327253 | 9.92E-05 | 1.05727 | 3.10% |
| rs7302200 | Asthma (adult onset) | 26582 | 300671 | 0.0884 | 327253 | 0.000183 | 0.934094 | 3.50% |
| rs1059513 | Asthma (adult onset) | 26582 | 300671 | 0.0884 | 327253 | 0.000218 | 1.11949 | 4.50% |
| rs28635831 | Asthma (adult onset) | 26582 | 300671 | 0.0884 | 327253 | 9.88E-05 | 1.05109 | 3% |
| rs4771332 | Asthma (adult onset) | 26582 | 300671 | 0.0884 | 327253 | 0.00013 | 0.942927 | 3.20% |
| rs7183955 | Asthma (adult onset) | 26582 | 300671 | 0.0884 | 327253 | 0.000121 | 1.06955 | 3.30% |
| rs72743461 | Asthma (adult onset) | 26582 | 300671 | 0.0884 | 327253 | 0.000268 | 0.911835 | 4.20% |
| rs35441874 | Asthma (adult onset) | 26582 | 300671 | 0.0884 | 327253 | 0.0002 | 1.08093 | 3.70% |
| rs3024655 | Asthma (adult onset) | 26582 | 300671 | 0.0884 | 327253 | 0.000158 | 0.882737 | 4.30% |
| rs112401631 | Asthma (adult onset) | 26582 | 300671 | 0.0884 | 327253 | 0.000147 | 0.811258 | 5.90% |
| rs117710327 | Asthma (adult onset) | 26582 | 300671 | 0.0884 | 327253 | 0.000215 | 1.15193 | 5.10% |
| rs891058 | Asthma (adult onset) | 26582 | 300671 | 0.0884 | 327253 | 9.18E-05 | 1.05126 | 3% |
| rs12470864 | Asthma (adult onset) | 26582 | 300671 | 0.0884 | 327253 | 0.000408 | 0.905753 | 5% |
| rs1041973 | Asthma (adult onset) | 26582 | 300671 | 0.0884 | 327253 | 0.000141 | 1.06917 | 3.30% |
| rs2381712 | Asthma (adult onset) | 26582 | 300671 | 0.0884 | 327253 | 0.00013 | 0.947059 | 3.10% |
| rs34290285 | Asthma (adult onset) | 26582 | 300671 | 0.0884 | 327253 | 0.000307 | 1.09969 | 4.50% |
| rs11088309 | Asthma (adult onset) | 26582 | 300671 | 0.0884 | 327253 | 0.000153 | 0.919155 | 3.60% |
| rs4491851 | Asthma (adult onset) | 26582 | 300671 | 0.0884 | 327253 | 0.00013 | 0.946879 | 3.10% |
| rs62296577 | Asthma (adult onset) | 26582 | 300671 | 0.0884 | 327253 | 0.000105 | 1.05321 | 3% |
| rs11715524 | Asthma (adult onset) | 26582 | 300671 | 0.0884 | 327253 | 0.000115 | 0.949974 | 3% |
| rs190438685 | Asthma (adult onset) | 26582 | 300671 | 0.0884 | 327253 | 0.000104 | 0.816126 | 5.10% |
| rs17454584 | Asthma (adult onset) | 26582 | 300671 | 0.0884 | 327253 | 0.000125 | 0.937118 | 3.20% |
| rs11742240 | Asthma (adult onset) | 26582 | 300671 | 0.0884 | 327253 | 0.000133 | 1.06323 | 3.20% |
| rs4099209 | Asthma (adult onset) | 26582 | 300671 | 0.0884 | 327253 | 0.000168 | 0.917777 | 3.70% |
| rs1898671 | Asthma (adult onset) | 26582 | 300671 | 0.0884 | 327253 | 0.000246 | 0.924602 | 3.90% |
| rs6866614 | Asthma (adult onset) | 26582 | 300671 | 0.0884 | 327253 | 0.000224 | 0.929859 | 3.70% |
| rs2338821 | Asthma (adult onset) | 26582 | 300671 | 0.0884 | 327253 | 0.000101 | 0.95189 | 3% |
| rs146003472 | Asthma (adult onset) | 26582 | 300671 | 0.0884 | 327253 | 0.000144 | 0.796106 | 6.30% |
| rs943689 | Asthma (adult onset) | 26582 | 300671 | 0.0884 | 327253 | 0.000204 | 1.07349 | 3.60% |
| rs13241235 | Asthma (adult onset) | 26582 | 300671 | 0.0884 | 327253 | 9.81E-05 | 1.05041 | 3% |
| rs57585717 | Asthma (adult onset) | 26582 | 300671 | 0.0884 | 327253 | 0.000109 | 0.926149 | 3.30% |
| rs7824278 | Asthma (adult onset) | 26582 | 300671 | 0.0884 | 327253 | 0.000153 | 1.06233 | 3.30% |
| rs66632892 | Asthma (adult onset) | 26582 | 300671 | 0.0884 | 327253 | 0.0001 | 0.946701 | 3% |
| rs992969 | Asthma (adult onset) | 26582 | 300671 | 0.0884 | 327253 | 0.000393 | 1.11548 | 5.20% |
| rs11121240 | Asthma (childhood onset) | 13962 | 300671 | 0.0464 | 314633 | 0.000115 | 0.931352 | 3.10% |
| rs9662290 | Asthma (childhood onset) | 13962 | 300671 | 0.0464 | 314633 | 9.61E-05 | 0.932691 | 3% |
| rs12123821 | Asthma (childhood onset) | 13962 | 300671 | 0.0464 | 314633 | 0.000746 | 0.656525 | 26.40% |
| rs61816761 | Asthma (childhood onset) | 13962 | 300671 | 0.0464 | 314633 | 0.000991 | 0.47995 | 76.10% |
| rs1617333 | Asthma (childhood onset) | 13962 | 300671 | 0.0464 | 314633 | 0.000198 | 1.09812 | 3.50% |
| rs10158467 | Asthma (childhood onset) | 13962 | 300671 | 0.0464 | 314633 | 0.000231 | 0.896019 | 3.90% |
| rs12023876 | Asthma (childhood onset) | 13962 | 300671 | 0.0464 | 314633 | 0.000125 | 1.07987 | 3.10% |
| rs12750027 | Asthma (childhood onset) | 13962 | 300671 | 0.0464 | 314633 | 0.000114 | 0.869787 | 3.70% |
| rs835673 | Asthma (childhood onset) | 13962 | 300671 | 0.0464 | 314633 | 9.47E-05 | 0.931038 | 3% |
| rs10737105 | Asthma (childhood onset) | 13962 | 300671 | 0.0464 | 314633 | 0.000203 | 1.12268 | 3.80% |
| rs2765414 | Asthma (childhood onset) | 13962 | 300671 | 0.0464 | 314633 | 0.000126 | 1.09099 | 3.20% |
| rs1612986 | Asthma (childhood onset) | 13962 | 300671 | 0.0464 | 314633 | 0.000338 | 0.856328 | 5.10% |
| rs10995245 | Asthma (childhood onset) | 13962 | 300671 | 0.0464 | 314633 | 0.000107 | 0.931956 | 3% |
| rs10786050 | Asthma (childhood onset) | 13962 | 300671 | 0.0464 | 314633 | 0.000169 | 0.917752 | 3.40% |
| rs11593589 | Asthma (childhood onset) | 13962 | 300671 | 0.0464 | 314633 | 0.000115 | 1.07221 | 3% |
| rs2052690 | Asthma (childhood onset) | 13962 | 300671 | 0.0464 | 314633 | 0.000106 | 0.928894 | 3.10% |
| rs10836538 | Asthma (childhood onset) | 13962 | 300671 | 0.0464 | 314633 | 0.000137 | 1.08321 | 3.20% |
| rs479844 | Asthma (childhood onset) | 13962 | 300671 | 0.0464 | 314633 | 0.000285 | 0.895698 | 4% |
| rs11236797 | Asthma (childhood onset) | 13962 | 300671 | 0.0464 | 314633 | 0.000886 | 0.823406 | 9.80% |
| rs12365699 | Asthma (childhood onset) | 13962 | 300671 | 0.0464 | 314633 | 0.000231 | 1.14126 | 4.20% |
| rs705700 | Asthma (childhood onset) | 13962 | 300671 | 0.0464 | 314633 | 0.000244 | 0.90236 | 3.80% |
| rs3122929 | Asthma (childhood onset) | 13962 | 300671 | 0.0464 | 314633 | 0.000395 | 0.876303 | 4.90% |
| rs11065979 | Asthma (childhood onset) | 13962 | 300671 | 0.0464 | 314633 | 0.000156 | 1.08502 | 3.30% |
| rs188074962 | Asthma (childhood onset) | 13962 | 300671 | 0.0464 | 314633 | 0.000244 | 1.11246 | 3.90% |
| rs7975763 | Asthma (childhood onset) | 13962 | 300671 | 0.0464 | 314633 | 0.00011 | 1.08807 | 3.90% |
| rs912425 | Asthma (childhood onset) | 13962 | 300671 | 0.0464 | 314633 | 0.000111 | 1.08376 | 3.10% |
| rs1887704 | Asthma (childhood onset) | 13962 | 300671 | 0.0464 | 314633 | 0.000196 | 0.907394 | 3.60% |
| rs17103286 | Asthma (childhood onset) | 13962 | 300671 | 0.0464 | 314633 | 0.000137 | 0.926643 | 3.20% |
| rs1885013 | Asthma (childhood onset) | 13962 | 300671 | 0.0464 | 314633 | 0.000168 | 1.09787 | 3.40% |
| rs1655558 | Asthma (childhood onset) | 13962 | 300671 | 0.0464 | 314633 | 0.000161 | 0.919987 | 3.30% |
| rs11071559 | Asthma (childhood onset) | 13962 | 300671 | 0.0464 | 314633 | 0.000314 | 1.18496 | 5.30% |
| rs72743461 | Asthma (childhood onset) | 13962 | 300671 | 0.0464 | 314633 | 0.000541 | 0.836304 | 6.90% |
| rs12935657 | Asthma (childhood onset) | 13962 | 300671 | 0.0464 | 314633 | 0.000366 | 1.15436 | 5% |
| rs3785356 | Asthma (childhood onset) | 13962 | 300671 | 0.0464 | 314633 | 0.000236 | 0.896086 | 3.90% |
| rs2066844 | Asthma (childhood onset) | 13962 | 300671 | 0.0464 | 314633 | 0.000106 | 0.853589 | 3.80% |
| rs61584523 | Asthma (childhood onset) | 13962 | 300671 | 0.0464 | 314633 | 9.75E-05 | 1.18425 | 3.90% |
| rs4795399 | Asthma (childhood onset) | 13962 | 300671 | 0.0464 | 314633 | 0.001655 | 1.30283 | 23.70% |
| rs11658582 | Asthma (childhood onset) | 13962 | 300671 | 0.0464 | 314633 | 0.000187 | 0.912738 | 3.50% |
| rs4792846 | Asthma (childhood onset) | 13962 | 300671 | 0.0464 | 314633 | 0.000154 | 1.08451 | 3.30% |
| rs7209400 | Asthma (childhood onset) | 13962 | 300671 | 0.0464 | 314633 | 0.000146 | 0.924102 | 3.20% |
| rs1893380 | Asthma (childhood onset) | 13962 | 300671 | 0.0464 | 314633 | 0.000142 | 0.92337 | 3.20% |
| rs12965763 | Asthma (childhood onset) | 13962 | 300671 | 0.0464 | 314633 | 0.000147 | 0.909338 | 3.40% |
| rs4574025 | Asthma (childhood onset) | 13962 | 300671 | 0.0464 | 314633 | 0.000181 | 0.915721 | 3.40% |
| rs12964116 | Asthma (childhood onset) | 13962 | 300671 | 0.0464 | 314633 | 0.00022 | 0.773634 | 6.40% |
| rs892225 | Asthma (childhood onset) | 13962 | 300671 | 0.0464 | 314633 | 0.00013 | 0.925896 | 3.20% |
| rs2918302 | Asthma (childhood onset) | 13962 | 300671 | 0.0464 | 314633 | 0.000127 | 0.903596 | 3.40% |
| rs113660049 | Asthma (childhood onset) | 13962 | 300671 | 0.0464 | 314633 | 0.000121 | 0.925415 | 3.10% |
| rs117710327 | Asthma (childhood onset) | 13962 | 300671 | 0.0464 | 314633 | 0.000203 | 1.20548 | 4.90% |
| rs3856439 | Asthma (childhood onset) | 13962 | 300671 | 0.0464 | 314633 | 0.000246 | 1.11341 | 3.90% |
| rs1962588 | Asthma (childhood onset) | 13962 | 300671 | 0.0464 | 314633 | 0.0001 | 0.922422 | 3.10% |
| rs72823641 | Asthma (childhood onset) | 13962 | 300671 | 0.0464 | 314633 | 0.001 | 1.34076 | 18.70% |
| rs143326447 | Asthma (childhood onset) | 13962 | 300671 | 0.0464 | 314633 | 0.000105 | 0.902426 | 3.30% |
| rs7582757 | Asthma (childhood onset) | 13962 | 300671 | 0.0464 | 314633 | 0.0001 | 0.937076 | 3% |
| rs10187276 | Asthma (childhood onset) | 13962 | 300671 | 0.0464 | 314633 | 0.000215 | 1.11605 | 3.80% |
| rs34290285 | Asthma (childhood onset) | 13962 | 300671 | 0.0464 | 314633 | 0.000597 | 1.19778 | 7.30% |
| rs13035466 | Asthma (childhood onset) | 13962 | 300671 | 0.0464 | 314633 | 9.49E-05 | 0.935764 | 3% |
| rs2766667 | Asthma (childhood onset) | 13962 | 300671 | 0.0464 | 314633 | 0.000105 | 1.07946 | 3.10% |
| rs2738783 | Asthma (childhood onset) | 13962 | 300671 | 0.0464 | 314633 | 0.00011 | 0.919593 | 3.20% |
| rs5758364 | Asthma (childhood onset) | 13962 | 300671 | 0.0464 | 314633 | 0.000135 | 1.09741 | 3.30% |
| rs35570272 | Asthma (childhood onset) | 13962 | 300671 | 0.0464 | 314633 | 0.000229 | 0.903966 | 3.70% |
| rs1806656 | Asthma (childhood onset) | 13962 | 300671 | 0.0464 | 314633 | 0.000126 | 1.08202 | 3.20% |
| rs2955118 | Asthma (childhood onset) | 13962 | 300671 | 0.0464 | 314633 | 0.000116 | 1.08175 | 3.10% |
| rs7625643 | Asthma (childhood onset) | 13962 | 300671 | 0.0464 | 314633 | 0.000104 | 0.934908 | 3% |
| rs7626218 | Asthma (childhood onset) | 13962 | 300671 | 0.0464 | 314633 | 0.000146 | 1.08332 | 3.20% |
| rs73192661 | Asthma (childhood onset) | 13962 | 300671 | 0.0464 | 314633 | 0.000374 | 0.881349 | 4.70% |
| rs56328339 | Asthma (childhood onset) | 13962 | 300671 | 0.0464 | 314633 | 0.00012 | 1.10129 | 3.30% |
| rs4916534 | Asthma (childhood onset) | 13962 | 300671 | 0.0464 | 314633 | 9.91E-05 | 1.12664 | 3.40% |
| rs5743618 | Asthma (childhood onset) | 13962 | 300671 | 0.0464 | 314633 | 0.00063 | 1.21008 | 8% |
| rs230495 | Asthma (childhood onset) | 13962 | 300671 | 0.0464 | 314633 | 1.00E-04 | 0.936226 | 3% |
| rs45613035 | Asthma (childhood onset) | 13962 | 300671 | 0.0464 | 314633 | 0.000276 | 0.833266 | 5.40% |
| rs6846348 | Asthma (childhood onset) | 13962 | 300671 | 0.0464 | 314633 | 0.000132 | 1.08507 | 3.20% |
| rs16903574 | Asthma (childhood onset) | 13962 | 300671 | 0.0464 | 314633 | 0.000193 | 0.839851 | 4.60% |
| rs12657787 | Asthma (childhood onset) | 13962 | 300671 | 0.0464 | 314633 | 0.000325 | 0.849237 | 5.30% |
| rs1837253 | Asthma (childhood onset) | 13962 | 300671 | 0.0464 | 314633 | 0.000513 | 0.846361 | 6.40% |
| rs2299012 | Asthma (childhood onset) | 13962 | 300671 | 0.0464 | 314633 | 0.000449 | 0.839763 | 6.30% |
| rs42403 | Asthma (childhood onset) | 13962 | 300671 | 0.0464 | 314633 | 0.000184 | 0.869917 | 4.10% |
| rs11135015 | Asthma (childhood onset) | 13962 | 300671 | 0.0464 | 314633 | 9.59E-05 | 0.934697 | 3% |
| rs9391997 | Asthma (childhood onset) | 13962 | 300671 | 0.0464 | 314633 | 0.000131 | 0.928512 | 3.10% |
| rs6910879 | Asthma (childhood onset) | 13962 | 300671 | 0.0464 | 314633 | 0.000309 | 0.810391 | 6.30% |
| rs55974914 | Asthma (childhood onset) | 13962 | 300671 | 0.0464 | 314633 | 9.97E-05 | 0.911341 | 3.20% |
| rs62408233 | Asthma (childhood onset) | 13962 | 300671 | 0.0464 | 314633 | 0.000251 | 1.11297 | 3.90% |
| rs9372120 | Asthma (childhood onset) | 13962 | 300671 | 0.0464 | 314633 | 0.000157 | 0.904475 | 3.50% |
| rs55743914 | Asthma (childhood onset) | 13962 | 300671 | 0.0464 | 314633 | 0.000173 | 0.904782 | 3.50% |
| rs6927172 | Asthma (childhood onset) | 13962 | 300671 | 0.0464 | 314633 | 0.000121 | 1.08976 | 3.20% |
| rs12531500 | Asthma (childhood onset) | 13962 | 300671 | 0.0464 | 314633 | 0.000224 | 1.10309 | 3.70% |
| rs6954667 | Asthma (childhood onset) | 13962 | 300671 | 0.0464 | 314633 | 0.000102 | 0.931182 | 3% |
| rs4722758 | Asthma (childhood onset) | 13962 | 300671 | 0.0464 | 314633 | 0.000216 | 0.887831 | 3.90% |
| rs4917131 | Asthma (childhood onset) | 13962 | 300671 | 0.0464 | 314633 | 9.86E-05 | 1.08637 | 3.10% |
| rs2202750 | Asthma (childhood onset) | 13962 | 300671 | 0.0464 | 314633 | 0.000252 | 1.11173 | 3.90% |
| rs4319131 | Asthma (childhood onset) | 13962 | 300671 | 0.0464 | 314633 | 0.000109 | 1.07054 | 3% |
| rs13277355 | Asthma (childhood onset) | 13962 | 300671 | 0.0464 | 314633 | 0.000181 | 1.10297 | 3.50% |
| rs340931 | Asthma (childhood onset) | 13962 | 300671 | 0.0464 | 314633 | 0.000207 | 0.905216 | 3.60% |
| rs7848215 | Asthma (childhood onset) | 13962 | 300671 | 0.0464 | 314633 | 0.000928 | 0.796999 | 12.30% |
| rs274943 | Asthma (childhood onset) | 13962 | 300671 | 0.0464 | 314633 | 0.000139 | 1.07957 | 3.20% |
| rs150707349 | Asthma (childhood onset) | 13962 | 300671 | 0.0464 | 314633 | 0.000101 | 1.21721 | 4.20% |
| rs10118244 | Asthma (childhood onset) | 13962 | 300671 | 0.0464 | 314633 | 9.80E-05 | 1.21258 | 4.10% |
| rs850637 | Asthma (childhood onset) | 13962 | 300671 | 0.0464 | 314633 | 0.000133 | 1.06413 | 3% |
| rs5953283 | Asthma (childhood onset) | 13962 | 300671 | 0.0464 | 314633 | 0.000131 | 1.06446 | 3% |

**Table S2. Heterogeneity and pleiotropy tests for instrumental variables in the analysis of asthma, adult-onset asthma, childhood-onset asthma, and the age of asthma diagnosis with delirium, before and after outlier removal.**

Before outliers removal:

| Exposure | Outcome | Heterogeneity | |  | Pleiotropy | |
| --- | --- | --- | --- | --- | --- | --- |
|  |  | Q statistic (IVW) | *P* value |  | MR-Egger Intercept | *P* value |
| Asthma | Delirium | 14.49 | 0.41 |  | 0.05 | 0.16 |
| Asthma (adult onset) |  | 41.38 | 0.54 |  | -0.04 | 0.11 |
| Asthma (childhood onset) |  | 105.34 | 0.24 |  | 0.0005 | 0.97 |
| Age asthma diagnosed |  | 22.47 | 0.43 |  | 0.05 | 0.04 |

After outliers removal:

| Exposure | Outcome | Heterogeneity | |  | Pleiotropy | |
| --- | --- | --- | --- | --- | --- | --- |
|  |  | Q statistic (IVW) | *P* value |  | MR-Egger Intercept | *P* value |
| Asthma | Delirium | 14.49 | 0.41 |  | 0.05 | 0.16 |
| Asthma (adult onset) |  | 41.38 | 0.54 |  | -0.04 | 0.11 |
| Asthma (childhood onset) |  | 105.34 | 0.24 |  | 0.0005 | 0.97 |
| Age asthma diagnosed |  | 21.67 | 0.42 |  | 0.06 | 0.06 |

**Table S3. MR-PRESSO test results for the analysis of asthma, adult-onset asthma, childhood-onset asthma, and the age of asthma diagnosis with delirium.**

| **Exposure** | **Outcome** | **Raw** | |  | **Outlier corrected** | | **Global P** | **Number of outliers** | **Distortion P** |
| --- | --- | --- | --- | --- | --- | --- | --- | --- | --- |
|  |  | **OR (CI%)** | **P** |  | **OR (CI%)** | **P** |  |  |  |
| Asthma | Delirium | 1.05 ( 0.83 - 1.34 ) | 0.68 |  | / | / | 0.44 | / | / |
| Asthma (adult onset) |  | 1.18 (0.84 - 1.65) | 0.35 |  | / | / | 0.427 | / | / |
| Asthma (childhood onset) |  | 1.03 (0.95 - 1.12) | 0.51 |  | / | / | 0.239 | / | / |
| Age asthma diagnosed |  | 1.04 (0.86 - 1.26) | 0.70 |  | / | / | 0.55 | / | / |

**Table S4.** Heterogeneity and pleiotropy tests for instrumental variables in the analysis of delirium with asthma, adult-onset asthma, childhood-onset asthma, and the age of asthma diagnosis.

| **Exposure** | **Outcome** | **Heterogeneity** | |  | **Pleiotropy** | |
| --- | --- | --- | --- | --- | --- | --- |
|  |  | **Q statistic (IVW)** | ***P* value** |  | **MR-Egger Intercept** | ***P* value** |
| Delirium | Asthma | 4.92 | 0.43 |  | -3.23E-02 | 0.21 |
|  | Asthma (adult onset) | 7.32 | 0.20 |  | 0.022 | 0.12 |
|  | Asthma (childhood onset) | 5.48 | 0.36 |  | 0.014 | 0.43 |
|  | Age asthma diagnosed | 2.87 | 0.58 |  | 0.007 | 0.50 |

**Table S5.** MR-PRESSO test results for the analysis of delirium with asthma, adult-onset asthma, childhood-onset asthma, and the age of asthma diagnosis.

| **Exposure** | **Outcome** | **Raw** | |  | **Outlier corrected** | | **Global P** | **Number of outliers** | | **Distortion P** |
| --- | --- | --- | --- | --- | --- | --- | --- | --- | --- | --- |
|  |  | **OR (CI%)** | **P** | |  | **OR (CI%)** | **P** |  |  |  |
| Delirium | Asthma | 1.04（0.98-1.11） | 0.18 |  | / | / | 0.37 | / | | / |
|  | Asthma (adult onset) | 0.98 (0.95 - 1.02) | 0.34 |  | / | / | 0.25 | / | | / |
|  | Asthma (childhood onset) | 1.01 (0.97 - 1.06) | 0.61 |  | / | / | 0.38 | / | | / |
|  | Age asthma diagnosed | 1.00 (0.98 - 1.02) | 0.94 |  | / | / | 0.60 | / | | / |
