# Supplementary material for: Age‐Related Genetic Causal Association Between Asthma and Delirium: A Bidirectional Two‐Sample Mendelian Randomization
Source: Brain Behav. 2026 Jan 28;16(2):e71198. doi: 10.1002/brb3.71198 (PMC12848525; doi:10.1002/brb3.71198)

**Figure S1. Scatter plots of SNP effects for asthma on delirium.** A. Age of asthma diagnosis to delirium; B. Adult-onset asthma to delirium; C. Childhood-onset asthma to delirium.


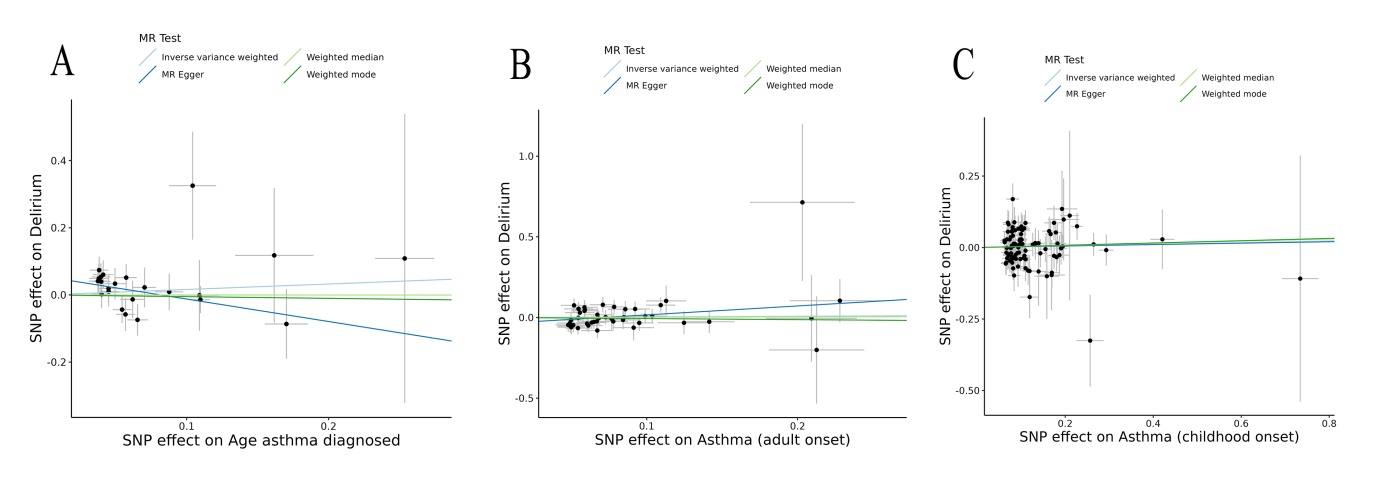


**Figure S2.** **Funnel plots of single-SNP MR for asthma on delirium.** A. Age of asthma diagnosis to delirium; B. Adult-onset asthma to delirium; C. Childhood-onset asthma to delirium.


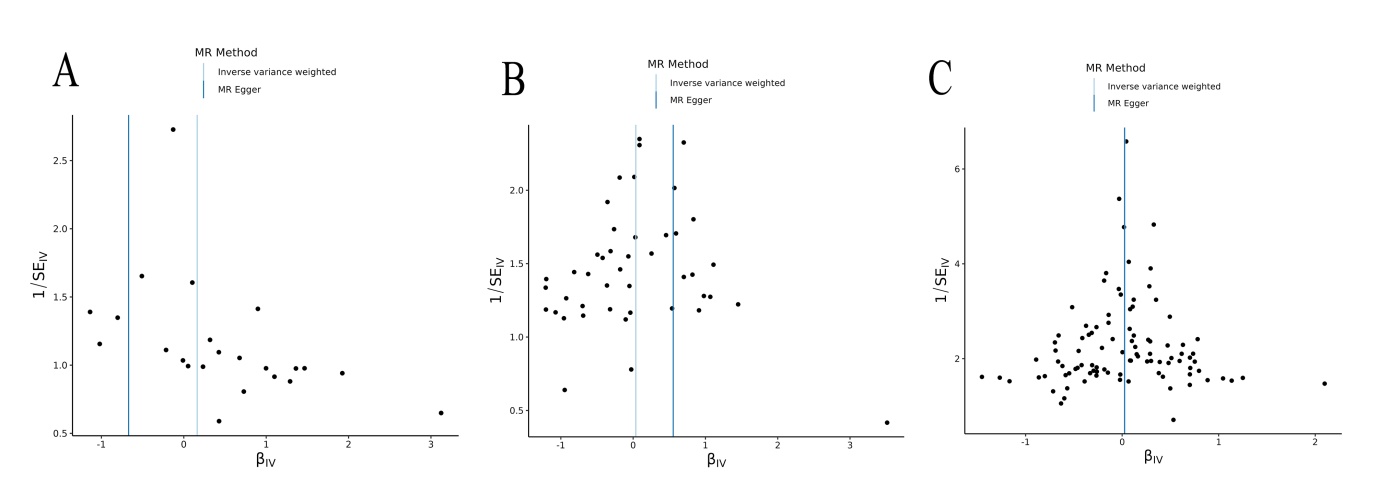


**Figure S3.** **Leave-one-out sensitivity analysis for asthma on delirium.** A. Age of asthma diagnosis to delirium; B. Adult-onset asthma to delirium; C. Childhood-onset asthma to delirium.


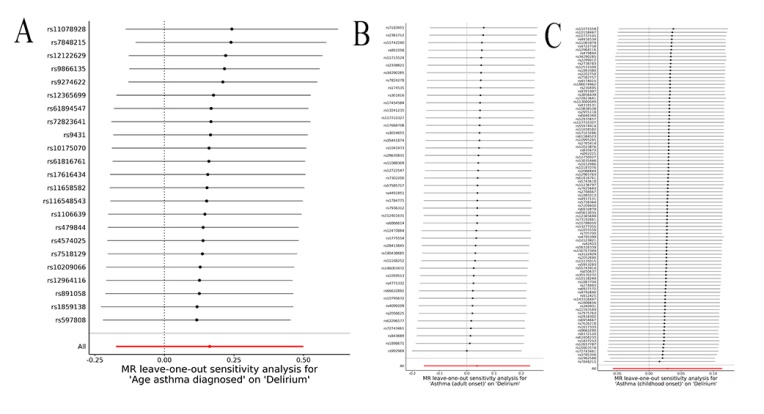


**Figure S4.** **Scatter plots of SNP effects for delirium on asthma.** A. Delirium to age of asthma diagnosis; B. Delirium to adult-onset asthma; C. Delirium to childhood-onset asthma.


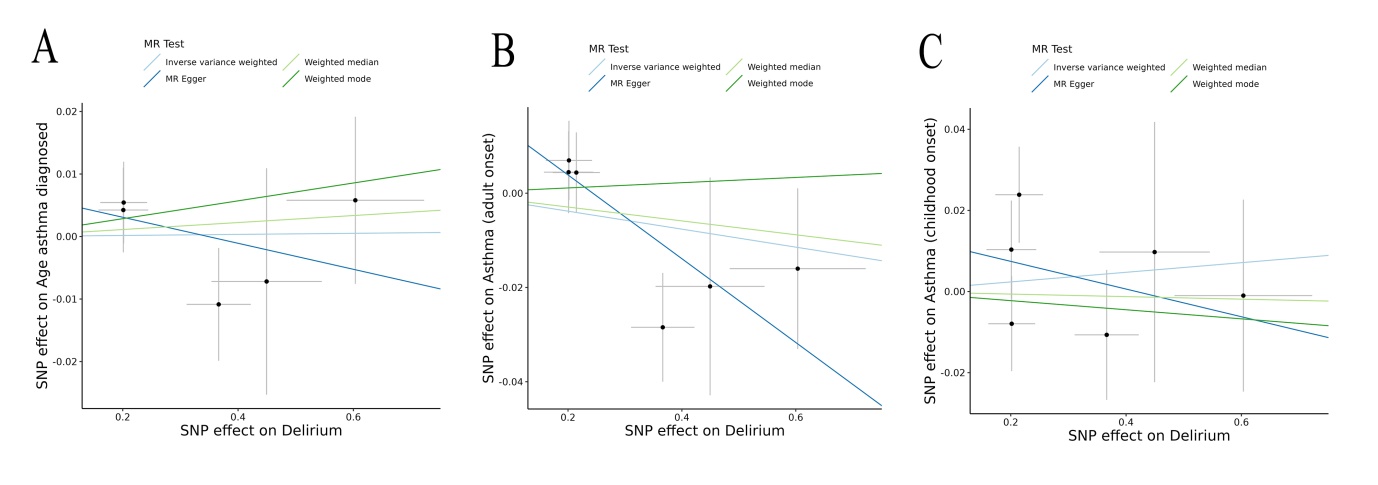


**Figure S5. Funnel plots of single-SNP MR for delirium on asthma.** A. Delirium to age of asthma diagnosis; B. Delirium to adult-onset asthma; C. Delirium to childhood-onset asthma.


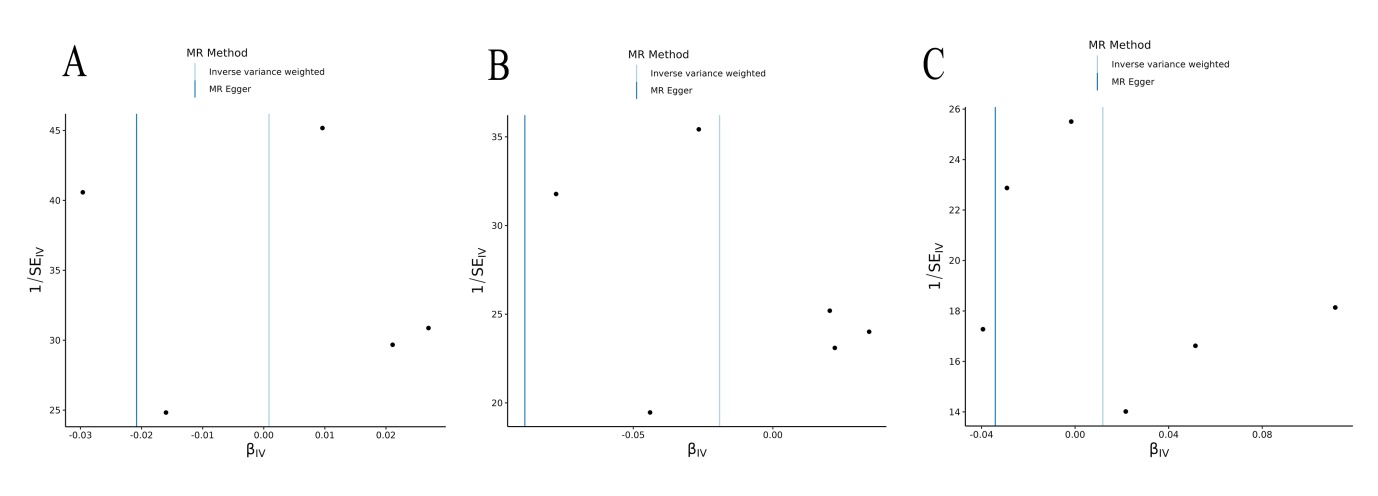


**Figure S6.** **Leave-one-out sensitivity analysis for delirium on asthma.** A. Delirium to age of asthma diagnosis; B. Delirium to adult-onset asthma; C. Delirium to childhood-onset asthma.


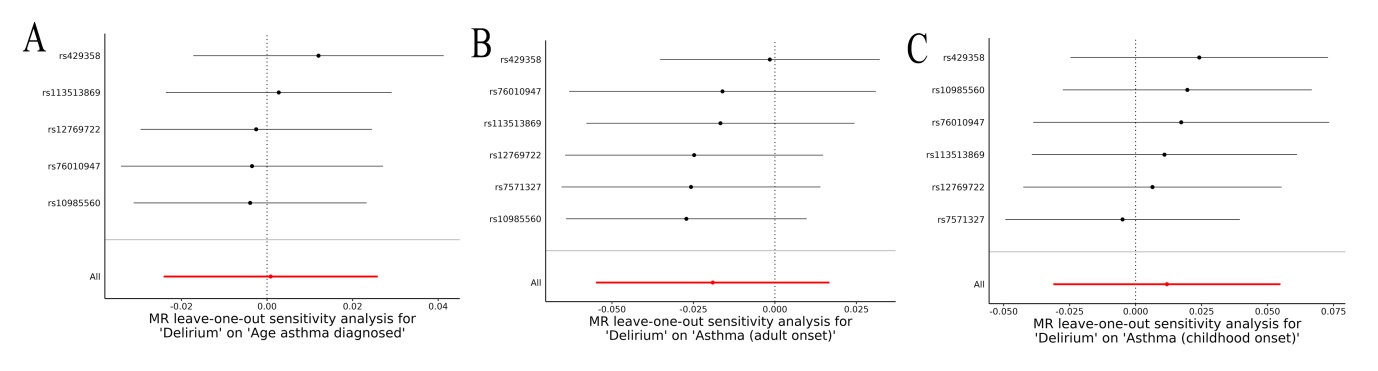

Supplement: Supplementary file 2 — Figure S1 Scatter plots of SNP effects for asthma on delirium. (A) Age of asthma diagnosis to delirium; (B) Adult‐onset asthma to delirium; and (C) Childhood‐onset asthma to delirium. Figure S2 Funnel plots of single‐SNP MR for asthma on delirium. (A) Age of asthma diagnosis to delirium; (B) Adult‐onset asthma to delirium; and (C) Childhood‐onset asthma to delirium. Figure S3 Leave‐one‐out sensitivity analysis for asthma on delirium. (A) Age of asthma diagnosis to delirium; (B) Adult‐onset asthma to delirium; and (C) Childhood‐onset asthma to delirium. Figure S4 Scatter plots of SNP effects for delirium on asthma. (A) Delirium to age of asthma diagnosis; (B) Delirium to adult‐onset asthma; and (C) Delirium to childhood‐onset asthma. Figure S5 Funnel plots of single‐SNP MR for delirium on asthma. (A) Delirium to age of asthma diagnosis; (B) Delirium to adult‐onset asthma; and (C) Delirium to childhood‐onset asthma. Figure S6 Leave‐one‐out sensitivity analysis for delirium on asthma. (A) Delirium to age of asthma diagnosis; (B) Delirium to adult‐onset asthma; and (C) Delirium to childhood‐onset asthma. [file BRB3-16-e71198-s001.docx]
